# A Single-Pass Type I Membrane Protein from the Apicomplexan Parasite *Cryptosporidium parvum* with Nanomolar Binding Affinity to Host Cell Surface

**DOI:** 10.3390/microorganisms9051015

**Published:** 2021-05-08

**Authors:** Tianyu Zhang, Xin Gao, Dongqiang Wang, Jixue Zhao, Nan Zhang, Qiushi Li, Guan Zhu, Jigang Yin

**Affiliations:** 1 Key Laboratory for Zoonosis Research of the Ministry of Education, College of Veterinary Medicine, Institute of Zoonosis, Jilin University, Changchun 130062, China; zhangty80@163.com (T.Z.); gaoxin18@mails.jlu.edu.cn (X.G.); wdq19@mails.jlu.edu.cn (D.W.); n_zhang@jlu.edu.cn (N.Z.); qiushili_jlu@126.com (Q.L.); 2 Peking-Tsinghua Center for Life Sciences and Academy for Advanced Interdisciplinary Studies, Peking University, Beijing 100871, China; 3 Department of Pediatric Surgery, First Hospital of Jilin University, Changchun 130021, China; jixue@jlu.edu.cn; 4 Hospital of Stomatology, Jilin University, Changchun 130041, China

**Keywords:** *Cryptosporidium parvum*, T-cell immunomodulatory protein (TIP) homolog, type I membrane protein, adhesion protein, binding kinetics, apparent dissociation constant

## Abstract

*Cryptosporidium parvum* is a globally recognized zoonotic parasite of medical and veterinary importance. This parasite mainly infects intestinal epithelial cells and causes mild to severe watery diarrhea that could be deadly in patients with weakened or defect immunity. However, its molecular interactions with hosts and pathogenesis, an important part in adaptation of parasitic lifestyle, remain poorly understood. Here we report the identification and characterization of a *C. parvum* T-cell immunomodulatory protein homolog (CpTIPH). CpTIPH is a 901-aa single-pass type I membrane protein encoded by cgd5_830 gene that also contains a short *Vibrio, Colwellia, Bradyrhizobium* and *Shewanella* (VCBS) repeat and relatively long integrin alpha (ITGA) N-terminus domain. Immunofluorescence assay confirmed the location of CpTIPH on the cell surface of *C. parvum* sporozoites. In congruence with the presence of VCBS repeat and ITGA domain, CpTIPH displayed high, nanomolar binding affinity to host cell surface (i.e., *K*_d(App)_ at 16.2 to 44.7 nM on fixed HCT-8 and CHO-K1 cells, respectively). The involvement of CpTIPH in the parasite invasion is partly supported by experiments showing that an anti-CpTIPH antibody could partially block the invasion of *C. parvum* sporozoites into host cells. These observations provide a strong basis for further investigation of the roles of CpTIPH in parasite-host cell interactions.

## 1. Introduction

*Cryptosporidium parvum* is a zoonotic protozoan parasite that causes gastroenteritis in a wide range of mammals including humans and a number of farm animals [1]. The impact of this parasite on human, animal and environmental health also makes it a good model under the One Health concept [2,3,4]. The life cycle of *C. parvum* starts after a host ingests oocysts that release sporozoites in the intestinal tract to invade epithelial cells [5]. Upon attachment of a sporozoite onto a targeted epithelial cell, host cell membrane at the infection site will start to grow upwards to cover the sporozoite that also undergoes morphological changes from banana-shape to a small round trophozoite. Each trophozoite will be fully contained within the host cell-derived membrane, termed parasitophorous vacuole membrane (PVM) on top of the intestinal epithelial cell, and undergo a merogonic development to form a meront that releases merozoites to invade new host cells. After two or more rounds of merogony, merozoites may start gametogenesis to form micro- and macro-gametes that fertilize to form zygotes. Zygotes will develop into mature oocysts containing four sporozoites before being excreted into environment.

In the life cycle development, the motile stages of *C. parvum* (i.e., sporozoites, merozoites and gametes) need to interact with extracellular matrix on the surface of host cells during gliding, attachment and invasion. In these biological processes, parasite surface proteins with extracytoplasmic domains are first line of molecules directly interacting with host cells, playing important roles in adaptation to the parasitic lifestyle. Therefore, the study of *C. parvum* surface proteins is important in potentially delineating the molecular interactions between the parasite and host cells. Currently, a number of *Cryptosporidium* surface proteins have been investigated at various molecular, biochemical and cellular levels. Examples include some mucin-like proteins, a number of thrombospondin type I domain (TSP1)-containing proteins and various immunodominant surface antigens [1,6,7,8]. These proteins were shown to be involved in interacting with host cells, while their exact roles and contributions to the parasite invasion remain to be fully elucidated.

**Figure 1:**
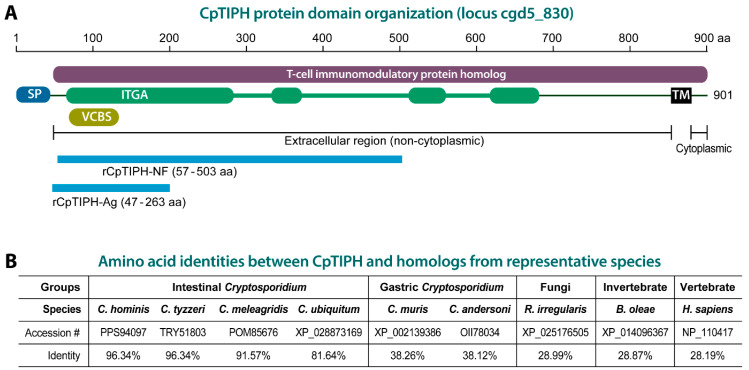
*Cryptosporidium parvum* T-cell immunomodulatory protein homolog (CpTIPH) domain organization (**A**) and amino acid identities with orthologs from other *Cryptosporidium* species, and selected fungal and animal species (**B**). Abbreviations: SP, signal peptide; IGTA, integrin alpha domain; VCBS, *Vibrio, Colwellia, Bradyrhizobium* and *Shewanella* (VCBS) repeat; TM, transmembrane domain; rCpTIPH-NF, recombinant CpTIPH N-terminal fragment used for binding assays; rCpTIPH-Ag, recombinant CpTIPH fragment used as an antigen for producing rabbit polyclonal antibodies. Full names for listed fungal and animal species: *Bactrocera oleae, Rhizophagus irregularis* and *Homo sapiens*.

In the present study, we aimed to discover novel surface proteins in *C. parvum* by focusing on secretory type I membrane proteins defined by the presence of a non-cytoplasmic domain that would be exposed to the extracellular side after trafficking to cytoplasmic membranes. By datamining the *C. parvum* genomes, we identified a unique type I molecule that contained an N-terminal signal peptide (SP) and a single transmembrane domain close to the C-terminus and was a homolog to T-cell immunomodulatory proteins (Figure 1). This protein also contained domains known for an adhesion property. We then performed experiments to confirm that this protein was a surface membrane protein in the parasite sporozoites, and capable of binding to the host cell surface with nanomolar binding affinity. Our data suggested that this membrane protein was involved in interacting with host cells during the invasion of *C. parvum* into host cells.

## 2. Materials and Methods

### 2.1. Identification of Cytoplasmic Membrane Proteins from C. parvum

To discover potential membrane proteins with significant surface exposure from *C. parvum*, we datamined the parasite genomes at the CryptoDB (https://cryptodb.org/, accessed on 7 May 2021) and identified a number of type I membrane proteins containing a signal peptide (SP) at the N-terminus and a single transmembrane (TM) domain near C-terminus. Among them, one gene product was homologous to T-cell immunomodulatory protein (TIP) family proteins (CryptoDB GeneID: cgd5_830; GenBank accession number: XM_626036) (Figure 1A). This *C. parvum* TIP homolog, designated as CpTIPH, contained orthologs that were highly conserved only within the intestinal *Cryptosporidium* species. Because nothing was known for this type I membrane protein, we decided to perform experiments on this parasite protein to provide a first set of data on the primary molecular and biological features.

### 2.2. The Parasite and In Vitro Cell Culture

*C. parvum* oocysts (gp60 subtype IIaA17G2R1) were originally purchased from Waterborne, Inc. (New Orleans, LA, USA) and propagated in-house by infecting calves. Oocysts were purified using a standard sucrose/CsCl gradient centrifugation protocol [9], and stored in 2.5% potassium dichromate at 4 °C until use. The oocysts used in all experiments were less than three months old. Prior to experiments, oocysts were surface sterilized by suspension in 4% (*v*/*v*) sodium hypochlorite on ice for 10 min, followed by three or more washes in PBS.

HCT-8 and CHO-K1 were purchased from Chinese Academy of Sciences Shanghai Branch and cultured in vitro in RPMI-1640 or Ham’s h F12 medium containing 10% bovine fetal serum (FBS) at 37 °C under 5% CO_2_ atmosphere. HCT-8 is a human intestinal epithelial cell line derived from an ileocecal colorectal adenocarcinoma patient (ATCC # CCL-244) and used to host the growth of *C. parvum* in vitro and to detect protein binding to host cell surface. CHO-K1 (ATCC # CCL-61) is an epithelial-like cell line derived as a subclone from a parental Chinese hamster ovary cell line and used as an additional cell line in cell surface protein binding experiments.

In vitro *C. parvum* infection assays were performed as described [10,11]. Briefly, HCT-8 cells were seeded into 96-well plates and allowed to grow overnight until cell monolayers reached ~90% confluence. Clean oocysts were added into the plates (10^4^ oocysts/well) and allowed for excystation and invasion for 2 h. After three washes with culture medium without FBS, the parasite was allowed to grow for 16 h or a specified time. At desired time points, cell lysates were prepared for quantitation of the parasite loads or levels of gene transcript by qRT-PCR as described below.

For preparing samples from intracellularly developing parasite for microscopic assays, HCT-8 cells were seeded into 24-well plates containing round glass coverslips and allowed to grow overnight until they reached ~90% confluence. Oocysts were added into plates for excystation and invasion for 2 h. After washes with culture medium, the parasite was allowed to grow for 22 h. Cell monolayers were fixed for immunofluorescence microscopic examination as described below.

### 2.3. Quantitative RT-PCR (qRT-PCR) Analysis of the CpTIPH Gene Expression

The relative levels of *CpTIPH* (cgd5_830) gene transcript during the parasite life cycle were detected by qRT-PCR using a pair of previously reported primers (5′-CTA TTT GGT TTG GGA AAG ACG A-3′ and 5′-TGA GTG TTT GGA ATG AGA CCT G-3′) [12]. The levels of *C. parvum* 18S rRNA were also detected using primers (5′-TAG AGA TTG GAG GTT GTT CCT-3′) and (5′-CTC CAC CAA CTA AGA ACG GCC-3′) for normalization as described [12,13]. Total RNA samples were extracted from oocysts, sporozoites and intracellular parasites infecting HCT-8 cells for various times using iScript qRT-PCR sample preparation reagent (lysis buffer) (Bio-Rad Laboratories, Hercules, CA, USA); qRT-PCR was carried out using HiScript^®^ II One-Step qRT-PCR SYBR^®^ Green Kit (Vazyme Biotech, Nanjing, China) as described [11].

Briefly, each 20 μL of reaction mixture contained 200 nM of each primer, 1 μL of One Step SYBR Enzyme Mix, 10 μL SYBR Green Mix, 0.4 μL ROX Reference Dye 1 (50×), 0.2 ng of total RNA from oocysts or sporozoites, or 15.0 ng of total RNA from infected cells. The mixtures were incubated at 50 °C for 3 min to synthesize cDNA, and then heated at 95 °C for 30 s to inactivate the reverse transcriptase, followed by 40 thermal cycles of PCR amplification (95 °C for 10 s, 60 °C for 30 s) on a Step One Plus thermal cycler (Thermo Fisher, Waltham, MA, USA). At least two technical replicated qRT-PCR reactions were performed for each sample. The relative levels of cgd5_830 gene transcript were calculated using a 2^−∆∆CT^ formula as described [13].

### 2.4. Expression of an N-Terminal Fragment of CpTIPH as a Recombinant Protein for Host Cell-Binding Assays

For cell surface binding assays, an N-terminal fragment of CpTIPH containing ITGA and VCBS domains (amino acids 57 to 503) was expressed as a recombinant GST-fusion protein (marked as rCpTIPH-NF in Figure 1). Corresponding DNA fragment (1341-bp) was amplified by PCR from total genomic DNA isolated from *C. parvum* oocysts using a TIANamp Stool DNA Kit (TIANGEN, Beijing, China) and primers 5′-GGA TCC TTT AAT TTC CCT GA-3′ and 5′-GAA TTC ATT GTT TTT CTG AGA ATG CT-3′ (restriction sites underlined). Thermal cycling used the following conditions: 95 °C for 5 min, followed by 35 cycles at 94 °C for 45 s, 55 °C for 45 s and 72 °C for 90 s, and a final extension at 72 °C for 10 min.

The PCR products were purified using a Gel/PCR Extraction Kit (BIOMIGA, San Diego, CA, USA) and cloned into a pGEX-4T-1 vector (Invitrogen) for expression as a glutathione-S-transferase (GST)-fusion protein in the BL21(DE3) strain of *Escherichia coli* (TIANGEN, Beijing, China). Recombinant protein was purified by an affinity chromatography using glutathione-sepharose 4B following manufacturer’s protocol (GE Healthcare, Stockholm, Sweden). The purity and molecular weight were evaluated by SDS-PAGE, followed by Coomassie Blue staining. This GST-fusion protein containing 447 aa N-terminal fragment without signal peptide was designated as GST-CpTIPH-NF.

### 2.5. Antibody Production and Affinit Purification

For generating CpTIPH-specific antibody, a 648 bp fragment encoding CpTIPH amino acids 47 to 263 was amplified from genomic DNA by PCR using primers 5′-GGA TCC AAC TCT TCA GGC ATA A-3′ and 5′-GAA TTC TTT GTG GAC TTT AAT GG-3′ (restriction sites are underlined). PCR thermal cycling and product purification followed the same protocols as described above. Purified PCR fragment was cloned into pET-28a vector (Novagen, Dusseldorf, Germany) and expressed in the BL21(DE3) strain of *E. coli*. The His-tagged recombinant protein as an antigen (marked as His-CpTIPH-Ag in Figure 1A) was purified using His GraviTrap columns according to the manufacturer’s instructions (GE Healthcare).

Two pathogen-free (SPF) rabbits were immunized with His-CpTIPH-Ag to produce polyclonal antibodies by a standard protocol [14]. Briefly, rabbits were immunized five times via a standard immunization protocol with two-week intervals. The first injection used 400 μg of recombinant protein mixed with an equal volume of Freund’s complete adjuvant, while the subsequent four injections used 300 μg of recombinant protein with Freund’s incomplete adjuvant. Pre-immune sera were collected as a negative control. IgG was affinity-purified with Protein A Sepharose 4 Fast Flow (GE Healthcare) according to the manufacturer’s protocol.

### 2.6. Western Blot Analysis

Freshly excysted sporozoites were prepared by an in vitro excystation protocol as reported [15]. Briefly, oocysts were incubated with PBS (pH 7.4) containing 0.75% taurodeoxycholic acid and 0.25% trypsin at 37 °C for 1 h, followed by addition of an equal volume of PBS containing 10% bovine serum albumin (BSA) to neutralize trypsin. Free sporozoites were collected and washed three times by centrifugation with PBS. Sporozoites were lysed in radioimmunoprecipitation assay (RIPA) buffer containing 1% Triton X-100 and protease inhibitor cocktail for eukaryotes (Sigma-Aldrich, St. Louis, MO, USA) on ice overnight. The lysates were heated in a reducing sample buffer for 5 min at 100 °C and subjected to sodium dodecyl sulfate polyacrylamide gel electrophoresis (10% SDS-PAGE).

Proteins were transferred onto nitrocellulose membranes [16]. The blots were incubated for 1 h in a blocking buffer containing 5% BSA in TBST (50 mM Tris-HCl, pH 7.5; 150 mM NaCl and 0.05% Tween-20), followed by incubation with purified primary antibody (1:250 dilution in 5% BSA in TBST) against CpTIPH for 1 h, five washes with TBST (5 min each), incubation with horseradish peroxidase (HRP)-conjugated goat anti-rabbit IgG antibody (ABclonal, Wuhan, China; 1:10,000 dilution) for 1 h, and five washes with TBST (5 min each). The blots were developed using an enhanced chemiluminescence reagent and visualized in a LAS 4000 mini luminescent image analyzer (GE Healthcare). All procedures were conducted at room temperature unless specified.

### 2.7. Indirect Immunofluorescence Assay (IFA) Labeling of CpTIPH

*C. parvum* sporozoites were prepared by an excystation protocol described above, fixed with 4% formaldehyde for 30 min, washed with PBS (pH 7.2) and applied onto poly-L-lysine coated glass slides. After standing for 2 h, slides were gently rinsed with PBS to remove unattached sporozoites and permeabilized with cold methanol:acetone (*v*:*v* = 1:1) for 5 min at −20 °C for 5 min. Intracellular parasites obtained by infecting HCT-8 cell monolayers on coverslips for 24 h were fixed with 4% formaldehyde for 30 min. After 3 washes with PBS, cells were permeabilized with PBS containing 0.1% Triton X-100 and 0.05% SDS for 5 min for subsequent IFA labeling.

Disrupted sporozoites were prepared by a hypotonic treatment of sporozoites with 0.2× PBS for 5 min, followed by a 5-min high-speed vortex. After centrifugation at 20,000× *g* for 10 min, pellets were resuspended in PBS, fixed with 4% paraformaldehyde for 30 min, applied onto poly-L-lysine coated glass slides and washed for 3 times with PBS for subsequent IFA labeling.

All fixed and permeabilized samples were blocked with 1% FBS in PBS (FBS-PBS) for 30 min, followed by incubation with affinity-purified anti-CpTIPH antibody (1:50 dilution in 5% FBS-PBS) overnight at 4 °C. Samples were washed with 1% FBS-PBS for 5 times (5 min each), followed by incubation with Alexa 488-labeled goat anti-rabbit antibody (1:2000 dilution in 1% FBS-PBS) for 1 h and 4 washes with 1% FBS-PBS [15]. After an additional wash with PBS, samples were mounted on glass slides in a SlowFade mounting medium containing Hoechst 33342 (Thermo Scientific) for counterstaining of nuclei and examined by an Olympus BX53 research fluorescence microscope.

### 2.8. In Vitro CpTIPH Binding Assays

Several assays were used to evaluate the binding of GST-CpTIPH-NF onto cultured mammalian cells. A binding kinetic assay was performed using a protocol similar to the enzyme-linked immunosorbent assay (ELISA) [17]. For binding to live cells, HCT-8 and CHO-K1 cells cultured to 100% confluence in 96-well plates were washed by serum-free RPMI 1640 medium and incubated with GST-CpTIPH-NF protein or GST-tag at various concentrations in RPMI 1640 medium containing 0.1% normal goat serum (NGS), 1 mM CaCl_2_, 1 mM MnCl_2_ at 4 °C for 1 h. For binding to fixed cells, HCT-8 and CHO-K1 cells were fixed with 1% glutaraldehyde in PBS for 30 min and quenched with 0.1 M glycine in PBS for 10 min, followed by 3 washes with RPMI 1640 and blocking with 5% NGS in RPMI 1640 at 4 °C for 4 h. After a wash with RPMI 1640, samples were incubated with GST-CpTIPH-NF or GST as described for experiments on live cells.

In both live and fixed cell-binding assays, cells were washed and fixed with ice-cold methanol for 10 min, and then washed with TBS, blocked with 2% NGS/TBS at 4 °C overnight. After a final wash with TBS buffer, CpTIPH-NF protein bound to host cells were detected by incubation with a monoclonal anti-GST antibody, biotinylated anti-mouse Fc IgG (Sigma-Aldrich), and streptavidin-conjugated alkaline phosphatase (Sigma-Aldrich) with 3 washes with TBS after each incubation step. The alkaline phosphatase (AP)-labeled specimens were developed with substrate p-nitrophenylphosphate (pNPP), followed by the measurement of absorption at 405 nm (OD_405_).

The binding of GST-CpTIPH-NF to host cell surface was also examined by an immunofluorescence-based microscopic and flowcytometric assays as described [18,19]. In microscopic assay, HCT-8 cells were cultured to confluence on 12-mm coverslips in 12-well plates, fixed with 4% paraformaldehyde for 1 h, washed with PBS and treated with 1% BSA in PBS for 1 h. Cells were then incubated with 200 nM GST-CpTIPH-NF or GST proteins overnight at 4 °C, followed by 3 washes, fixation with methanol for 10 min, 3 washings with PBS and blocking with 1% BSA-PBS for 1 h. Samples were incubated with mouse anti-GST (Tianjin Sungene Biotech, Tianjin, China) antibody for 1 h. After 3 washes, cells were incubated with Alexa 488-labeled goat anti-mouse antibody for 1 h. Fluorescence-labeled slides were mounted in a SlowFade mounting medium containing Hoechst. In these experiments, 1% BSA in PBS buffer or PBS was used in all incubations or washes.

In fluorescence activated cell sorting (FACS)-based flowcytometry assay [19], HCT-8 cells were washed with RPMI 1640 and dispersed with 500 μM EDTA in PBS for 10 min at 37 °C. Detached cells were washed with 2% FBS (2% FBS in PBS) by centrifugation at 500× *g* for three times. After washes, cells were incubated with 400 μL GST-CpTIPH-NF or GST at 7.5 μM for 2 h at 4 °C and washed with FACS buffer. Cells were then incubated with mouse anti-GST monoclonal antibody (TransGen; 1:1000 dilution) for 30 min at 4 °C. After 3 washes with 2% FBS, cells were incubated with Alexa 488-labeled goat anti-mouse antibody (1:1000 dilution) for 30 min at 4 °C, washed with FACS buffer and quantitated on a FACSCalibur system (BD Bioscience, Franklin Lakes, NJ, USA).

### 2.9. In Vitro Invasion Assay

The effect of anti-CpTIPH IgG on the invasion of *C. parvum* into HCT-8 cells was evaluated using an in vitro assay as described with minor modifications [20]. Briefly, HCT-8 cells were cultured in 96-well plates to ~90% confluence. Oocysts of *C. parvum* (2 × 10^4^/well) were pre-incubated with an anti-CpTIPH antibody at different concentrations for 30 min in RPMI 1640 medium containing 10% FBS at 37 °C, and then added to the wells to infect host cells for 2 h at 37 °C. Oocyst walls and free sporozoites were removed by exchanging the culture medium. Parasite-infected cells were then incubated at 37 °C for additional 16 h (i.e., total 18 h infection time). Mouse pre-immune IgG was used in parallel as a control. After incubation, cells were washed 3 times with RPMI 1640.

Parasite loads were evaluated by qRT-PCR detection of the relative levels of parasite 18S rRNA as described [11]. Briefly, total RNA was isolated from HCT-8 cells infected with *C. parvum* using an iScript qRT-PCR sample preparation reagent (lysis buffer) (Bio-Rad Laboratories, Hercules, CA); qRT-PCR reactions were performed using a one-step SYBR Green qRT-PCR kit (DBI Bioscience, Germany) with the following pairs of primers: Cp18S-1011F (5′-TTG TTC CTT ACT CCT TCA GCA C-3′) and Cp18S-1185R (5′-TCC TTC CTA TGT CTG GAC CTG-3′) for *C. parvum* 18S rRNA (Cp18S), and Hs18S-1F (5′-GGC GCCCCC TCG ATG CTC TTA-3′) and Hs18S-1R (5′-CCC CCG GCC GTC CCT CTT A-3′) for host cell 18S rRNA (Hs18S). Reaction mixtures were incubated at 42 °C for 30 min for synthesizing cDNA, heated at 95 °C for 2 min to deactivate the reverse transcriptase and then subjected to 40 thermal cycles of PCR amplification (95 °C for 10 s, 55 °C for 34 s and 72 °C for 30 s). At least three technical replicates were performed for each sample. The parasite loads were calculated using the empirical 2^−ΔΔCT^ formula.

### 2.10. Statistical Analysis

All experiments were performed independently for at least three times, and each contained at least three technical replicates. Results were expressed as the mean ± the standard error of the mean (SEM). Statistical analyses were performed using Prism program version 8 or above (GraphPad, San Diego, CA, USA). Statistical significance was determined with Student’s *t*-test.

## 3. Results

### 3.1. CpTIPH Is a Type I Transmembrane Protein with Homology to T-Cell Immunomodulatory Proteins

In search for potential adhesion proteins in *C. parvum*, we identified a single-pass type I membrane protein homologous to the family of T-cell immunomodulatory proteins (TIPs) based on InterProScan analysis (Figure 1A). The protein, designated here as CpTIPH, was encoded by a 2706-bp open-reading frame (ORF) predicting 901 amino acids (aa) that was annotated as “Signal peptide uncharacterized transmembrane protein” at CryptoDB (GeneID: cgd5_830) or “membrane associated protein with a transmembrane domain near C-terminus” at GenBank (accession number: XM_626036). CpTIPH was composed of an N-terminal signal peptide (SP) (1–31 aa) and a TIP homolog (IPR024881/PTHR13412) for virtually all the remaining sequence (45–898 aa). The TIP homolog also contained four regions of 215, 46, 34 and 62 aa with significant homology to an integrin alpha (ITGA) N-terminal domain superfamily (SSF69318; E-value = 1.7 × 10^−24^) and a *Vibrio, Colwellia, Bradyrhizobium* and *Shewanella* (VCBS) repeat (IPR013517 and pfam13517) (Figure 1A). CpTIPH contained a transmembrane domain close to the C-terminus and was predicted to be a type I plasma member protein with a long extracellular region (i.e., the majority of the sequence without SP) and a very short 22 aa cytoplasmic domain (Figure 1A).

CpTIPH have orthologs in the genome of all other *Cryptosporidium* species available at the CryptoDB (i.e., *C. hominis, C. tyzzeri, C. ubiquitum, C. meleagridis, C. muris* and *C. andersoni*), suggesting that this protein plays a common role in the *Cryptosporidium* genus. The conceptually translated protein sequence of CpTIPH share 81.6–96.3% and 38.1–38.2% amino acid identities to the orthologs from intestinal and gastric *Cryptosporidium* species, respectively (Figure 1B), indicating that *Cryptosporidium* TIPH proteins are highly divergent between intestinal and gastric species. CpTIPH is also highly divergent from homologs of non-alveolate species, such as those from fungi (e.g., *Rhizophagus irregularis*, 28.99% identity), invertebrates (e.g., *Bactrocera oleae*, 28.87%) and vertebrates (e.g., human *Homo sapiens*, 28.19%) (Figure 1B).

### 3.2. CpTIPH Is a Surface Membrane Protein in C. parvum Sporozoites and Expressed during the Parasite Life Cycle

**Figure 2:**
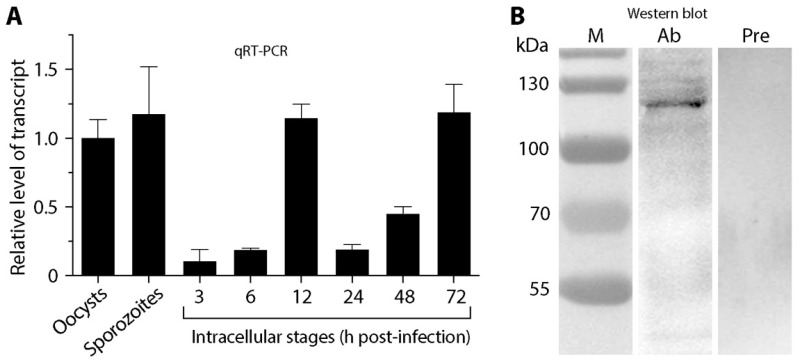
Detection of *CpTIPH* gene (cgd5-830) transcript and native protein. (**A**) Relative levels of *CpTIPH* gene at various developmental stages by qRT-PCR. (**B**) Native protein from sporozoite crude extracts by Western blot analysis using an affinity-purified polyclonal antibody. Lanes: M, protein marker; Ab, sample labeled with polyclonal antibody; Pre, sample labeled with pre-immune serum used as a negative control. Error bars represent the SEM of three biological replicates.

The *CpTIPH* gene transcript was detectable by qRT-PCR at all developmental stages of *C. parvum*, from oocysts, excysted sporozoites and intracellular parasites at various post-infection time points (Figure 2A). The relative levels of transcript were high in extracellular stages (oocysts and sporozoites) and intracellular parasite at 12 and 72 h post-infection (hpi) time points, in comparison with those at 3, 6, 24 and 48 hpi time points. The parasite at 3 and 6 hpi represented highly synchronized developing trophozoites and early stage of type I meronts after invasion. The much lower levels of transcript at 3 and 6 hpi time points (10.9–18.9% vs. oocysts) were suggestive that *CpTIPH* gene was much less active shortly after the parasite established infection.

In comparison with other protein-coding genes, *CpTIPH* was one of the lowly expressed genes in *C. parvum*. By mining an earlier microarray data [21], the transcript level of cgd5_830 was ranked at 2117 among 3806 genes in oocysts. In the RNA-seq-based transcriptomics datasets available in CryptoDB, the levels of cgd5_830 transcript were mostly at the bottom 25–50 percentiles. In an earlier proteomics analysis, CpTIPH was not among the 1237 proteins (~30% of the predicted proteome) detected in sporozoites [22].

To detect native CpTIPH protein, polyclonal antibodies were raised in rabbits against a recombinant protein. Using an affinity-purified antibody, CpTIPH protein was detected as a single major band in the crude extracts of *C. parvum* sporozoites by Western blot analysis (vs. no detectable bands using pre-immune serum) (Figure 2B). The molecular weight of the antibody-recognized major band (~120 kDa) was larger than that of predicted from protein sequence (i.e., 102.2 kDa), which might be due to the possible glycosylation that was common in *Cryptosporidium* surface adhesion proteins.

**Figure 3:**
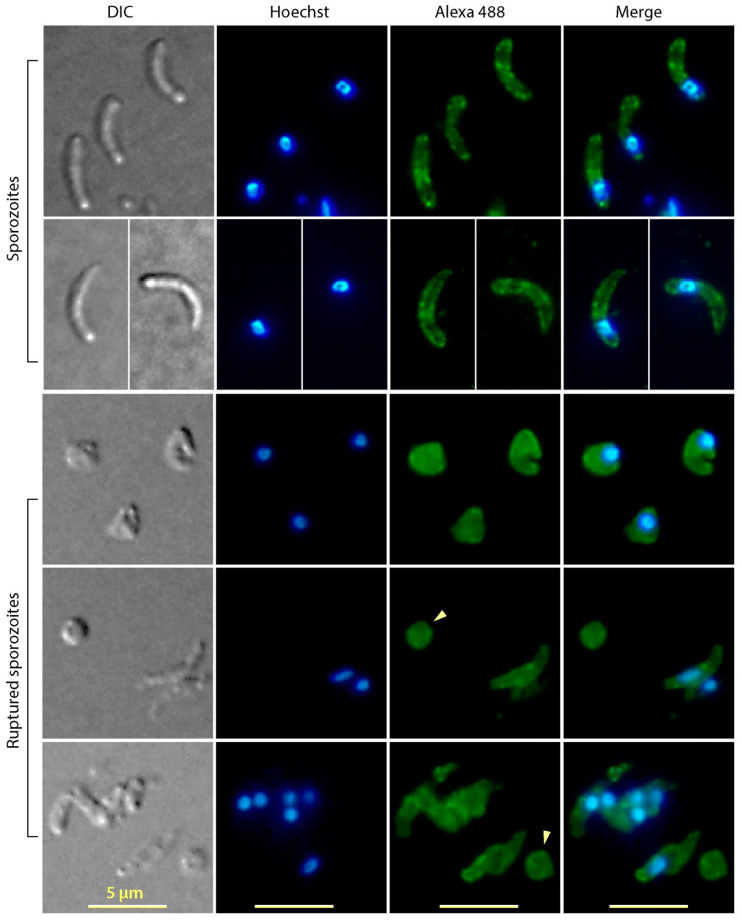
Immunofluorescence assay labeling of CpTIPH protein in intact and ruptured *Cryptosporidium parvum* sporozoites. Ruptured sporozoites were prepared by hypotonic treatment and vortex. DIC, differential interference contrast; Hoechst, nuclei counterstained using Hoechst. Arrowheads indicate fractured sporozoite membranes containing no nuclei.

**Figure 4:**
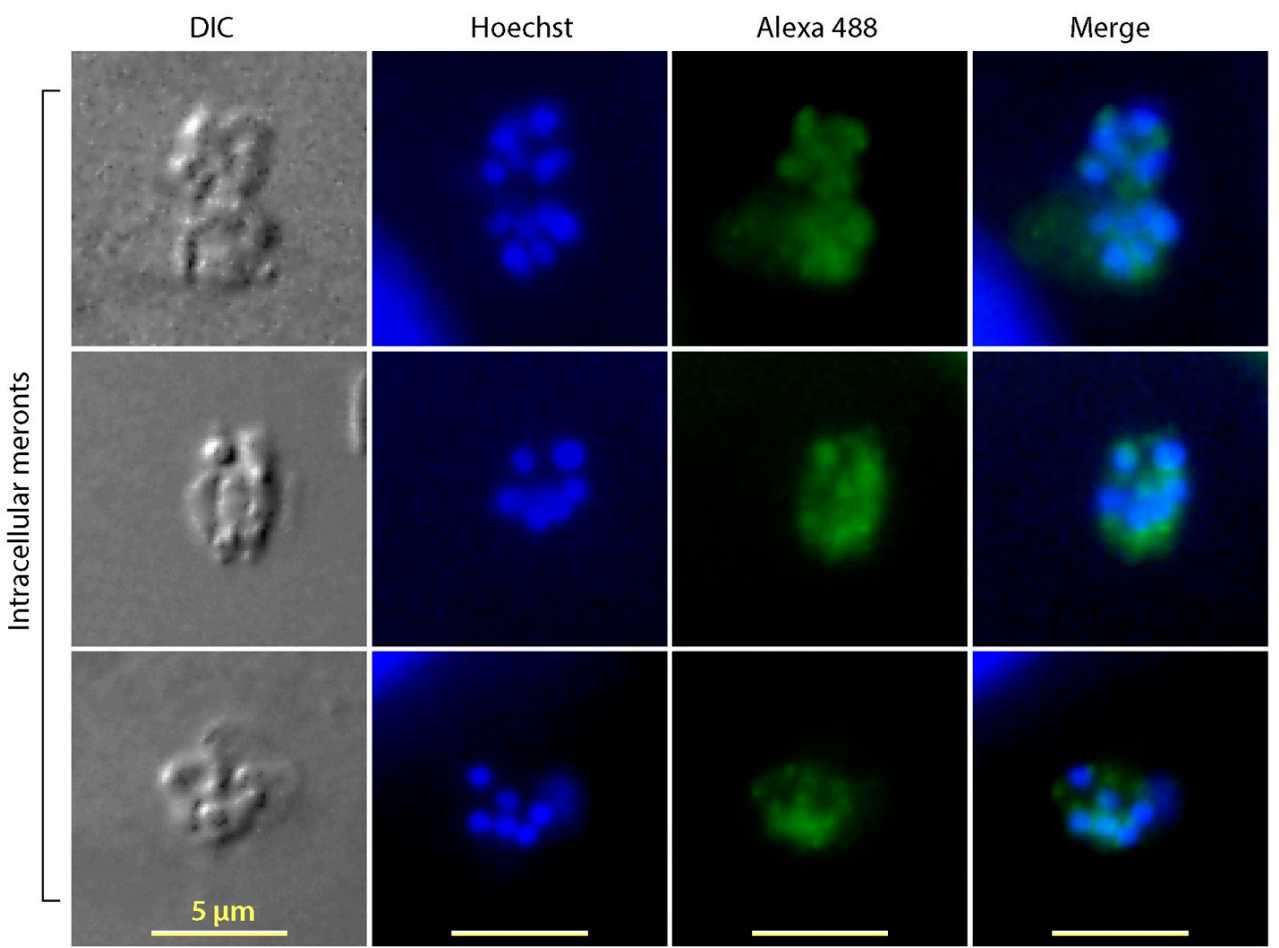
Immunofluorescence assay labeling of CpTIPH protein in intracellularly meronts of *Cryptosporidium parvum*. DIC, differential interference contrast; Hoechst, nuclei counterstained using Hoechst.

Immunofluorescence microscopic assay (IFA) detected CpTIPH protein in *C. parvum* sporozoites and meronts (Figure 3). In intact sporozoites, major granulated fluorescent signals from the surface were observed (Figure 3). The membrane association of CpTIPH was further confirmed by IFA on ruptured sporozoites prepared by vortex of sporozoites suspended in 0.2× PBS (Figure 3). The hypotonic treatment effectively disintegrated sporozoite integrity as indicated by the severe deformation of sporozoites and the loss of nuclei in some membrane pieces (Figure 4, arrowheads). Therefore, soluble proteins were likely lost and only membrane associated proteins could be labeled by IFA. These observations agreed with the prediction that CpTIPH was a type I cytoplasmic membrane protein.

Fluorescent signals were also detected in the intracellular meronts (Figure 4). Although constrained by the resolution of light microscopy for precisely defining the subcellular location of fluorescent signals inside the small meronts, the IFA apparently labeled the intracellular merozoites, but not on the parasitophorous vacuole membrane (PVM), which excluded that CpTIPH was a PVM protein.

### 3.3. CpTIPH Is Capable of Binding to Host Cell Surface with Nanomolar Binding Affinity

**Figure 5:**
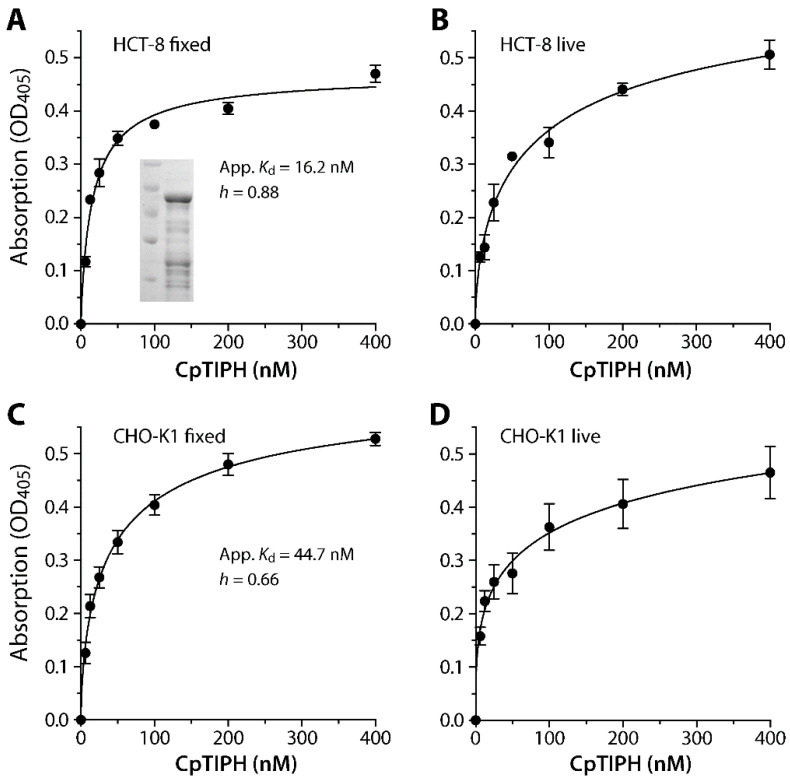
Binding kinetics of a recombinant CpTIPH protein N-terminal fragment (CpTIPH-NF) (inset) on fixed and live HCT-8 (**A**,**B**) and CHO-K1 (**C**,**D**) cells as determined by an ELISA-like protocol using an affinity-purified polyclonal antibody. Apparent dissociation constant (App. *K*_d_) values were determined for binding to fixed cells that reached to saturation (**A**,**C**). The binding followed a negative cooperative binding kinetics with Hill coefficient (***h***) values at 0.88 and 0.66 on the two cell lines. OD, optical density.

T-cell immunomodulatory proteins, ITGA domain and VCBS repeat were known for adhesion and interacting with other cell surface proteins. To test whether CpTIPH protein was able to interact with host cell surface, a fragment of CpTIPH containing the VCBS and ITGA domains was expressed as a recombinant GST-fusion protein (GST-CpTIPH-NF) for investigating its interaction with host cells. Heterogenous expression of recombinant protein was successful, and GST-CpTIPH-NF was shown as a single major band by SDS-PAGE at expected size (Figure 5A, inset). Some minor bands were present, likely a result of incomplete translation of foreign genes in *E. coli* due to different codon usages. These minor bands represented partial GST-CpTIPH-NF proteins, rather than contaminants of other proteins, as confirmed by Western blot analysis using an anti-GST antibody (data not shown).

The specific binding of GST-CpTIPH-NF to both live and fixed mammalian cells from two cell lines (i.e., HCT-8 and CHO-K1) was observed by a fluorescence assay using an anti-GST antibody (Figure 5). The binding of GST-CpTIPH-NF, with the subtraction of signals from GST-tag as a negative control, reached to saturation on fixed cells for both cell lines, but the binding was less saturated on live cells that was possibly due to the uptake of some GST-CpTIPH-NF protein by host cells. Therefore, we calculated the apparent dissociation constants (*K*_d(app)_) for binding of GST-CpTIPH-NF to the surface of fixed cells, which were 16.2 and 44.7 nM for HCT-8 and CHO-K1 cells, respectively (Figure 5A,C). There was ~2.7-fold difference between the two *K*_d(app)_ values, but they were both at the lower nM range, indicating high binding affinity of CpTIPH to host cell surface. The binding followed negative cooperative binding kinetics (i.e., Hill coefficient *h* = 0.88 and 0.66 for binding to fixed HCT-8 and CHO cells, respectively), implying the presence of competing ligands on the host cell surface.

**Figure 6:**
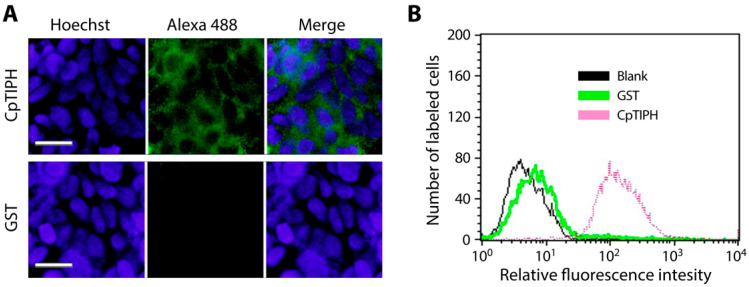
Detection of the binding of CpTIPH protein to the surface of fixed HCT-8 cells by immunofluorescence assay (IFA) (**A**) and fluorescence activated cell sorting (FACS) flowcytometry (**B**). In both assays, recombinant GST-CpTIPH-NF protein was incubated with fixed host cells, labeled with an anti-GST antibody and an Alexa Fluo 488-conjugated secondary antibody. GST-tag alone was used as a negative control. In IFA, host cell nuclei were counterstained by Hoechst.

The binding of CpTIPH on fixed host cells was further confirmed by IFA and FACS. In IFA using an anti-GST antibody, bright fuorescent signals were detected on the surface of fixed HCT-8 cells treated with GST-CpTIPH-NF protein, but not on cells treated with GST (Figure 6A). Similarly, FACS detected elevated levels of fluorescent signals from cells treated with GST-CpTIPH-NF, whereas signals from GST-treated cells remained comparable to those from cells receiving no treatment (Figure 6B).

### 3.4. Anti-CpTIPH Antibody Partially Blocked the C. parvum Infection

**Figure 7:**
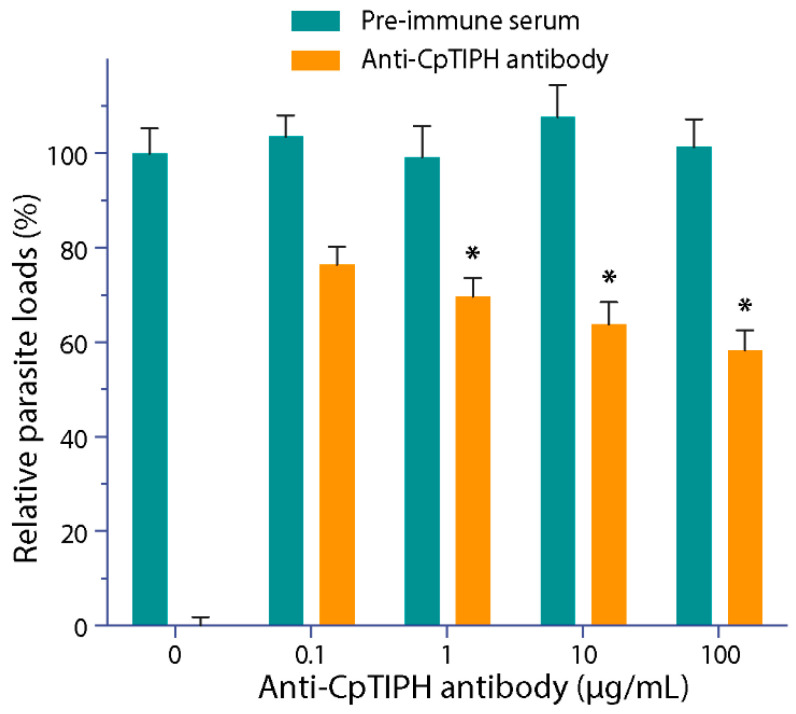
Effect of affinity-purified anti-CpTIPH polyclonal antibody on the parasite infection. In this assay, *C. parvum* oocysts were incubated with the antibody or pre-immune serum for 30 min, and then were incubated together with HCT-8 cells for 2 h. After washes, cells were allowed to grow for additional 16 h (total 18 h infection time), followed isolation of total RNA and qRT-PCR to determine the relative parasite loads. Data represent the mean ± SEM. * *p* < 0.05 (vs. control using pre-immune serum).

Since CpTIPH was found to be a surface membrane protein in the infectious sporozoite stage, we investigated the effect of blocking CpTIPH by the affinity-purified anti-CpTIPH antibody on the parasite infection. By incubating anti-CpTIPH antibody with *C. parvum* oocysts and cultured HCT-8 cells for 2 h during the invasion, followed by continuous parasite growth in an antibody-free medium for 16 h and a qRT-PCR assay, we observed a dose-dependent reduction of the parasite loads (Figure 7). Statistical significance was observed in groups treated with antibody concentrations at 1 to 100 μg/mL, in which the parasite loads were reduced by 30.2% to 42.7% (*p* < 0.05). These observations suggested that CpTIPH as a surface protein likely plays a role during the invasion of sporozoites into host cells.

## 4. Discussion

A number of proteins present on the surface of or discharged from the sporozoites or merozoites of *C. parvum* have been reported for their potential roles during the parasite invasion of host cells. Examples of the surface proteins included mucin-like proteins (e.g., GP900, GP60 (GP40/15), CpMuc4 and CpMuc5), TSP1 domain-containing proteins (e.g., TRAP-C1), lectin-like protein (CpClec), a circumsporozoite-like protein (CSL) and a number of other immunodominant antigens (e.g., P23, CP47, Cpa135, CPS-500), which were comprehensively described in several recent and earlier review articles (e.g., [1,7,8]). This study added a new member to the list by identifying and characterizing a new surface protein from the parasite belonging to the family of type I membrane proteins that are single-pass molecules targeted to the cytoplasmic membrane with a long extracellular N-terminal domain [23].

This parasite protein is a T-cell immunomodulatory protein homolog (TIPH), thus termed CpTIPH, which also contains a VBSC repeat and domains with weak homology to ITGA. TIPHs and ITGA homologs are widely present in eukaryotes [24,25,26], while VCBS repeat is present at high copy numbers in some species of the bacterial *Vibrio, Colwellia, Bradyrhizobium* and *Shewanella* (InterPro domain IPR010221) [27]. TIPHs, ITGA and VBSC repeat are known for their adhesion property involved in cell-to-cell interactions and have been identified in other apicomplexan parasites (e.g., *Toxoplasma* and *Plasmodium*) for their potential involvement in the attachment and invasion of parasites into host cells [25,26,28,29]. In the present study, we experimentally confirmed the binding affinity of the N-terminal half of the CpTIPH to host cells, and observed low nanomolar *K*_d_ binding affinity, suggesting that CpTIPH is an excellent cell surface binding protein.

As a type I membrane protein on the parasite cell surface, CpTIPH may serve as an adhesive molecule for sporozoites during their moving and gliding on the gastrointestinal epithelial surfaces, penetration of mucosal layers, attachment/invasion of host cells at the infection sites, and/or for anchoring parasite cells to surrounding molecules during intracellular development. We provided preliminary evidence on the involvement of CpTIPH in the parasite invasion by showing that an anti-CpTIPH antibody partially blocked the invasion of *C. parvum* sporozoites into host cells. This concept was also partially supported by the transcript levels that were higher in motile sporozoites, but much lower in stages containing no motile parasites (e.g., 3 and 6 hpi time points shortly after invasion) (Figure 2A). Nonetheless, the exact roles played by CpTIPH remain an intriguing question that is worth further investigation.

## 5. Conclusions

We identified a new member of the surface proteins from the apicomplexan parasite *C. parvum* and determined its binding affinity and kinetics on the host cell surface. These observations provide a strong basis to further investigate its role in the parasite–host cell interactions and to discover its binding ligand(s) on the host cells.
